# A swine model of severe chronic thromboembolic pulmonary hypertension induced by repeated pulmonary artery long suture injection

**DOI:** 10.3389/fcvm.2025.1736958

**Published:** 2026-01-16

**Authors:** Liliana Moreira-Costa, André Leite-Moreira, Rui Adão, David Laville, Marta Tavares-Silva, Isabel Miranda, Rui J. Cerqueira, Francisca C. Correia, Stéphanie Chanon, Aurélie Vieille-Marchiset, Frédéric Perros, Adelino F. Leite-Moreira, André P. Lourenço, Pedro Mendes-Ferreira

**Affiliations:** 1 RISE-Health, Department of Surgery and Physiology, Faculty of Medicine, University of Porto, Porto, Portugal; 2 São João Local Health Unit, Department of Anesthesiology, Porto, Portugal; 3 Department of Pharmacology and Toxicology, School of Medicine, Universidad Complutense de Madrid, Madrid, Spain; 4 CIBER Enfermedades Respiratorias (CIBERES), Madrid, Spain; 5 Laboratoire CarMeN, UMR INSERM U1060/INRA U1397, University of Lyon, Université Claude Bernard Lyon 1, Pierre-Bénite, France; 6 São João Local Health Unit, Department of Cardiology, Porto, Portugal; 7 São João Local Health Unit, Department of Cardiothoracic Surgery, Porto, Portugal; 8 Paris-Porto Pulmonary Hypertension Collaborative Laboratory (3PH), INSERM UMR_S 999, Université Paris-Saclay, Le Kremlin-Bicêtre, France

**Keywords:** chronic thromboembolic pulmonary hypertension, pulmonary circulation, right ventricle, swine model, vascular remodeling

## Abstract

**Introduction:**

Large animal models are key to translational research. Current models of chronic thromboembolic pulmonary hypertension (CTEPH) are rather complex, impractical and most fail to achieve a severe PH and right ventricular (RV) dysfunction phenotype. Our aim was to develop a plain large animal model of severe CTEPH with RV dysfunction.

**Methods:**

In 3 consecutive weeks, 2-month-old male pigs (∼25 kg) randomly underwent either left and right pulmonary artery (PA) injection of 15–30 15 cm #0 silk sutures (CTEPH, *n* = 9) or sham procedure (Sham, *n* = 6). Embolization was interrupted based on mean PA pressure (mPAP) elevation, cardiac output (CO) or systemic blood pressure decline, or complete obstruction on angiography. After 4 weeks of follow-up, we assessed echocardiography, biventricular pressure-volume (PV) hemodynamics, RV skinned cardiomyocyte, lung and RV histology, and RV gene expression and protein levels.

**Results:**

At terminal evaluation, mPAP was consistently higher in CTEPH compared with Sham (51 ± 3 vs. 20 ± 1 mmHg, respectively; *P* < 0.001) with reduced CO (4.2 ± 0.2 vs. 6.6 ± 0.9 L.min^−1^; *P* = 0.042). On angiography, most of the PA territory was obstructed, with cephalad redirection of flow. RV hypertrophy was clear on morphology, echocardiography and histology. CTEPH showed decreased RV ejection fraction (35 ± 2 vs. 51 ± 3%; *P* < 0.001), delayed relaxation, and end-diastolic stiffening but preserved ventricle-vascular coupling. Although fibrosis was not observed on histology and collagen chain expression was not upregulated in RV myocardium, skinned RV cardiomyocytes from CTEPH did show passive stiffening as well as higher active tension development. CTEPH RV showed altered gene expression of myosin heavy chains and B-type natriuretic peptide as well as decreased sarcoendoplasmic reticulum Ca^2+^-ATPase (SERCA2a) expression. Both SERCA2a and phosphorylated phospholamban protein levels were decreased as well. Medial hypertrophy was observed in distal arteries from the unobstructed right cranial lobe of CTEPH denoting flow-induced vasculopathy.

**Discussion:**

We developed a new and straightforward swine model of severe CTEPH with RV dysfunction by repeated injection of long sutures into the PA without the need for additional hits which may be valuable for preclinical research.

## 1 Introduction

Pulmonary hypertension (PH) is a chronic disease of the pulmonary vasculature characterized by a progressive increase in pulmonary vascular resistance (PVR). With an estimated worldwide prevalence of 1% (1), and growing incidence (2), it is diagnosed by mean pulmonary arterial (PA) pressure (mPAP) > 20 mmHg at rest on right heart catheterization (RHC) (3). Disease progression leads to right ventricle (RV) overload and ultimately failure. The most prevalent forms are due to left heart disease and lung disease and/or hypoxia (groups 2 and 3, respectively). These forms typically exhibit a more protracted clinical course, with progression and outcomes largely influenced by the underlying condition. In contrast, rarer forms such as pulmonary arterial hypertension (PAH, group 1) and chronic thromboembolic pulmonary hypertension (CTEPH, group 4) (4) tend to progress more rapidly due to intrinsic vascular remodeling or persistent vascular obstruction. Available therapies focus mainly on controlling dysregulated pulmonary vascular tone and delaying PVR increase and do not directly target the RV. Still, RV function remains the key determinant of outcome (5). Clinical outcomes remain suboptimal, warranting further research. Particularly in these rarer forms, experimental animal model research has fueled many of the therapeutic breakthroughs. Nevertheless, many of the findings from experimental research, most noticeably rodent models, have failed to translate into clinical practice (6), raising substantial concerns. Large animal models, most predominantly swine, that most closely resemble human physiology, are thus instrumental (7, 8), despite being limited by the need of specialized infrastructure and increased costs when compared to rodents. CTEPH, likely because of straightforward causation, has been the most common PH class emulated in large animal models. Still, emulating PH in CTEPH is challenging (9). Most literature reports have been unsuccessful, achieving less-than-severe PH even with very complex approaches that are rather impractical (10–12). Models are complex and time consuming (13–17), usually leading to mild phenotypes compared to humans (18). Embolization models are challenging because the RV tolerates acute PH poorly, and its failure is often irreversible. Therefore, a cautious, stepwise embolization strategy is essential. One of the simplest swine models consisted of alternating embolization of the distal branches of the left and right pulmonary arteries with microspheres, combined with embolization of more proximal arteries using short silk suture segments (3 cm in length). This procedure induced a significant, yet relatively mild and heterogeneous, pulmonary hypertension (14).

Assuming that using larger suture segments as drivers of macrovascular obstruction would better mimic the pathophysiology of CTEPH, we aimed to develop a new swine model of CTEPH with severe PH. The left and right PA of swine were alternately embolized using 15 cm long, 0 USP (0.35 mm) silk sutures once per week in 3 interventions with additional follow-up for 4 weeks. At follow-up, CTEPH animals showed severe PH, associated with RV dysfunction and pulmonary vascular remodeling similarly to the human condition. This new model closely mimics human CTEPH, representing a valuable tool in translational research in the field of PH and RV failure.

## 2 Methods

All animal procedures were conducted in accordance with the Guide for the Care and Use of Laboratory Animals (NIH Publication No. 85-23, revised 2011), approved by the Animal Ethics Committee of the Faculty of Medicine, University of Porto, and certified by the Portuguese National Authority for Animal Health—DGAV (Direção-Geral de Alimentação e Veterinária, certification no. 0421/000/000/2021, 2021-07-30 011706).

Fifteen uncastrated male pigs (Landrace×Large White; 25–30 kg; 2–3 months old) were obtained from a DGAV-licensed breeder (PTAH03). Animals were housed under standard conditions, fed daily, and had *ad libitum* access to drinking water. Following a 5-day quarantine, pigs were randomly assigned to one of two groups: (i) weekly alternate pulmonary artery (PA) embolization using silk sutures for 3 weeks, or (ii) sham procedures involving injection of an equal volume of saline. Animals were randomized in blocks of 2:3 (Sham:CTEPH).

**Figure 1:**
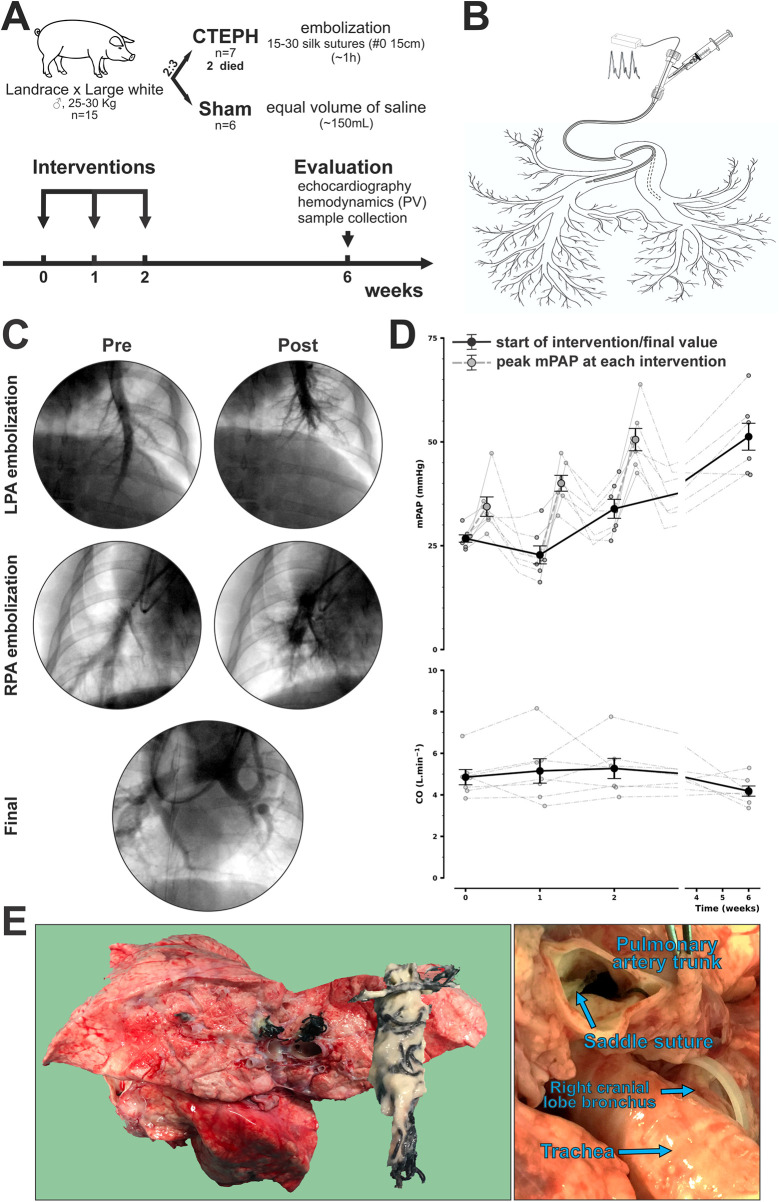
Study protocol and evolution of the pig chronic thromboembolic pulmonary hypertension (CTEPH) experimental model. **(A)** Study design; **(B)** schematic representation of suture embolization intervention, please note deployment of sutures preserves the right cranial lobe artery; **(C)** representative pulmonary artery (PA) angiograms before and after (Pre and Post, respectively) left and right PA embolization (LPA and RPA, respectively) and final angiography; **(D)** evolution of mean PA pressure (mPAP) and cardiac output (CO) in CTEPH and, finally, **(E)** macroscopic aspect of suture embolization. In **(E)**, the image on the left shows large arteries obstruction by sutures in a lung lobe and a suture plug dissected from a main pulmonary artery whereas the right image shows a saddle suture emboli in the main pulmonary trunk. In **(D)**, black lines, symbols and error bars represent mean ± error at the start of each intervention as well as final values before terminal hemodynamic evaluation while dashed grey lines and error bars illustrate mPAP rise at each intervention, individual animal point data scatters are presented as well. In **(C,D)** only data for CTEPH is presented. PV, pressure-volume.

Terminal evaluation was performed 4 weeks after the final intervention. The study design is illustrated on Figure 1A.

### 2.1 Animal preparation

Following overnight fasting, animals were premedicated with ketamine (15 mg·kg^−1^), midazolam (0.5 mg·kg^−1^), and azaperone (4 mg·kg^−1^). General anesthesia was induced with a propofol bolus (2 mg·kg^−1^) and maintained via continuous infusion (10–20 mg·kg^−1^·h^−1^). Analgesia was provided using fentanyl (3 µg·kg^−1^ bolus, followed by 2 µg·kg^−1^·h^−1^ infusion). Intravenous cefazolin (25 mg·kg^−1^) was administered as antibiotic prophylaxis.

Animals were orotracheally intubated and ventilated with room air at a tidal volume of 8–10 mL.kg^−1^, a respiratory rate of 15–20 breaths.min^−1^, adjusted to maintain normocapnia. Positive end-expiratory pressure was set at 5 cmH_2_O. An arterial catheter (Arterial Leadercath 20G, Vygon) was placed in a branch of the left or right superficial femoral artery for systemic blood pressure monitoring and recording (LabChart 8, ADInstruments). Continuous monitoring included peripheral oxygen saturation, capnography, body temperature, systemic blood pressure, and three-lead electrocardiography. Normothermia was maintained using a heating pad.

### 2.2 Intervention

An 8 Fr introducer sheath (Radifocus® Introducer, Terumo) was placed in the right internal jugular vein under ultrasound guidance. A Swan-Ganz thermodilution catheter (Edwards) was advanced into the PA, and baseline PA pressure (PAP) and cardiac output (CO) were recorded. A 0.035-inch, 260 cm guidewire was then introduced through the catheter's distal lumen and advanced into a distal PA segment.

The Swan-Ganz catheter was exchanged over the guidewire for a 6 Fr long sheath (Flexor® Shuttle® Guiding Sheath, Cook Medical), positioned in either the left or right PA. The sheath's cross-cut valve was replaced with a Y-connector, enabling simultaneous introduction of a 3.5 Fr tipped Mikro-Cath (Millar), equipped with a radiopaque marker band for continuous pressure monitoring, and injection of either contrast medium or silk sutures. The experimental setup is illustrated in Figure 1B.

Following baseline pulmonary angiography, 15–30 sterile silk sutures (size 0, 15 cm length) were injected through the PA sheath in animals assigned to the CTEPH group (final n = 7). Each suture was manually loaded into a 10 mL syringe and purged with high pressure for deployment into the pulmonary vasculature (as illustrated in Supplementary Video 1). Throughout the procedure PAP, CO, and systemic blood pressure were continuously monitored.

Suture injection was halted upon meeting any of the following criteria: mPAP exceeding 40, 45, or 50 mmHg during the first, second, or third intervention, respectively; a drop in CO; systemic hypotension (mean blood pressure—mBP -<60 mmHg); or angiographic evidence of complete vessel occlusion. The procedure lasted approximately 1 h per session.

The embolization protocol was repeated weekly for 3 consecutive weeks, beginning with the left PA and alternating sides thereafter. A sham group (n = 6) underwent right heart catheterization (RHC) with saline infusion at matched time points; the average volume used to deliver sutures in one session was estimated at 150 mL.

All procedures were conducted under normothermic and normocapnic conditions, with room air ventilation to minimize external influences on vascular tone. The first intervention was systematically performed in the left PA, selected due to its relatively smaller vascular territory. The right cranial lobe artery, originating craniolaterally at the proximal right PA (19), was systematically preserved (as detailed in Figure 1B).

Representative angiograms obtained before and after left and right PA embolization, as well as following the final intervention, are shown in Figures 1C,D illustrates the evolution of mean PAP and CO in CTEPH animals. Data from Sham is not presented for simplicity's sake.

### 2.3 Transthoracic echocardiography and invasive hemodynamic studies

Four weeks after the last intervention, animals were anesthetized and ventilated as described in the animal preparation section. Transthoracic echocardiography was performed using a cardiac probe (4v1c, Acuson Sequoia C512, Siemens). Measurements included: RV free wall thickness (RVWT), PA acceleration/ejection time (PAAT/PAET), left ventricle (LV) eccentricity index at early diastole in short axis (EI) and tricuspid annular plane systolic excursion (TAPSE). At least three stable cardiac cycles were averaged for all measurements.

Following echocardiography, 10 Fr introducer sheaths were placed in a femoral artery and vein for LV catheterization with a pressure-volume (PV) catheter (Ventri-Cath 507, Millar) and inferior vena cava (IVC) occlusion balloon deployment, respectively. Two additional 8 Fr introducer sheaths were placed in the right internal jugular vein for RHC, one for CO monitoring with thermodilution and another for PV catheter (Ventri-Cath 507, Millar). To allow simultaneous recording of left and right PV loops, each catheter was connected to a separate control unit (MPVS Ultra, Millar).

In a closed-chest percutaneous setup, thermodilution CO measurements were used for calibration of field inhomogeneity (α), parallel conductance was derived by central injection of 15% NaCl bolus (5–7 mL), and IVC occlusions were achieved using the occlusion balloon. Acquisitions were performed upon a 15 min stabilization period with ventilation suspended at end-expiration. Data was analyzed using custom Python scripts. End-systolic (ES) PV relationships (ESPVR) were modelled with simple linear fitting upon checking that quadratic terms were dismal, end-diastolic (ED) PV relationships (EDPVR) were modelled with exponential fitting including an asymptote. The time constant of isovolumic relaxation, *τ*, was derived by logistic fitting.

After ensuring adequate anesthetic depth with propofol (10 mg·kg^−1^) and fentanyl (3 µg·kg^−1^) bolus administration, sternotomy was performed, the thoracic aorta and inferior vena cava were lacerated for exsanguination, and the heart and lungs were harvested *en bloc* and washed. Transmural RV samples were collected from the trabecular free wall at the mid-ventricular level and were either snap-frozen in liquid nitrogen and stored at −80 °C or collected for histopathology (formalin-fixed paraffin embedded tissue). The non-obstructed cranial right lung lobe was dissected and perfused with 10% (v/v) buffered formalin for adequate fixation to assess flow-induced distal vasculopathy.

### 2.4 Morphometric and histological analysis

The right and left ventricles were dissected and weighed separately. For histological assessment, 3 µm-thick sections of the right ventricle (RV) were stained with hematoxylin and eosin (H&E) to quantify cardiomyocyte cross-sectional area (CSA), and with picrosirius red (PSR) to evaluate myocardial fibrosis. Cranial right lung lobe as well as lower lobe samples were formalin-fixed, and paraffin embedded; 3 µm-thick sections were obtained and stained with hematoxylin-eosin-saffron (HES) to assess distal arteriole remodeling and lung parenchyma modifications.

Digital images were acquired and analyzed using ImageJ software. For CSA quantification, a minimum of 60 cardiomyocytes per animal were measured in transverse cross-section at the level of the nucleus. Myocardial fibrosis was quantified as the percentage of PSR-positive (red) area, excluding background, across at least 10 sections per animal. Distal arteriole remodeling was assessed by calculating the percent wall thickness from a minimum of 10 arterioles per animal. Lung HES were also blindly analyzed by an experienced pathologist (DL) to assess other lung structural modifications on a qualitative basis.

### 2.5 *In vitro* studies in isolated RV skinned cardiomyocytes

RV skinned cardiomyocytes were isolated as previously described (20). In brief, samples were defrosted in cold relax solution [5.95 mM Na_2_ATP, 6.04 mM MgCl_2_.6H_2_O, 2.00 mM ethylene glycol tetra acetic acid (EGTA), 139.6 mM KCl, 10 mM imidazole, pH 7.0], mechanically disrupted and permeabilized with 0.1% Triton X-100. Using microscopic view (1X51, Olympus) and imaging software (VSL 900B, Aurora Scientific), single cardiomyocytes were attached to a force transducer and a length controller (403 A and 315 C-I, Aurora Scientific, respectively). Cell length was adjusted digitally (series 600 A digital controller, Aurora Scientific).

Cardiomyocyte stiffness was assessed through sarcomere length (SL)–passive tension (PT) relationships between 1.8 and 2.3 µm. Calcium-dependent active tension (AT) relationships were obtained at 2.2 µm SL in activating solutions with different Ca^2+^ concentrations [pCa: 5.0, 5.2, 5.4, 5.6, 5.8 and 6.0; diluted from a stock of 5.97 mM Na_2_ATP, 6.28 Mm MgCl_2_, 40.64 mM propionic acid, 100 mM N,N-Bis(2-hydroxyethyl)taurine, 7 mM Ca-EGTA and 14.5 mM phosphocreatine disodium salt hydrate, Na_2_PCr]. Passive force was measured through a “slack” test (cells were shortened to 80% of the initial length) in relax solution. AT was derived by subtracting passive force from total force. Experiments were carried out at 15 °C. Force was normalized for myocyte CSA, which was derived assuming an elliptical shape. A minimum of 5 cardiomyocytes were analyzed per animal.

### 2.6 Real-time quantitative PCR

Total RNA was extracted from 30 mg of frozen RV tissue using NZYol (Nzytech) following the manufacturer's instructions. RNA concentration and integrity were assessed by Nanodrop (Thermo Fisher Scientific®) and electrophoresis. cDNA (100 ng.µL^−1^) was synthesized using the SensiFAST™ cDNA Synthesis kit (Meridian Bioscience Inc®) and reactions were conducted in a T100 thermal cycler (Bio-Rad®). Real-time quantitative polymerase chain reaction (RT-qPCR) reactions were run in duplicate using SensiFAST™ SYBR® Hi-ROX Kit (Meridian Bioscience Inc®) in PikoReal™ Real-Time PCR System (Thermo Fisher Scientific®), according to manufacturer's instructions. Before gene expression quantification using the 2−ΔΔCT method, PCR efficiency of each gene including the internal control genes (glyceraldehyde-3-phosphate dehydrogenase, GAPDH, and 60S ribosomal protein L4, RPL4) was determined and it was ensured that they were identical. Target gene expression was normalized using the geometric mean of both housekeeping genes (21). No differences were observed in the expression of control genes. Data are presented relative to Sham expression. Primer sequences (Table 1) were designed in-house with Primer-BLAST (NCBI). Melting-curve analysis and primer-BLAST checks ensured amplicon specificity.

**Table 1:** Real-time quantitative PCR genes, primers and amplicons.

Gene	Primer sequence (5’ → 3’)	Amplicon size (bp)
ATP2A2	*fw:* TTGGCATCTTTGGGCAGG *rev:* ATTCAGGCAGGCTTCTCTCT	101
COL1A1	*fw:* AACATGGAGACAGGCGAGAC *rev:* CGCCGTACTCGAACTGGAA	145
COL3A1	*fw:* GAGGTAATGATGGTGCCCGA *rev:* CGGGTCCAACTTCACCCTTAG	108
EDN1	*fw:* TGGGTCAACACTCCAGAACAC *rev:* TGGCACACTGGCATCTATCC	124
GAPDH	*fw:* CATCATCCCTGCTTCTACCG *rev:* CCTGCTTCACCACCTTCTTG	179
MYH6	*fw:* AGCTCATGGCCACACTCTTC *rev:* TTGTTCAGATTCTCCCGGTGG	130
MYH7	*fw:* TACTTTGCTGTCATTGCCGC *rev:* TTGTCATTCCTAACGGTCTTGG	140
NPPB	*fw:* CAAGTCCTCCGGGGAATACG *rev:* ACCTCCTGAGCACATTGCAG	115
RPL4	*fw:* AATGTCACTTTGCCTGCTGT *rev:* TGGCTGTCTGTTGTTTTTGCG	93
RYR2	*fw:* TCCACTTCCAGGTCTTCAACT *rev:* GCCAACTCGCACTTTTTCTCC	135
TNF	*fw:* TGGCCCAAGGACTCAGATCAT *rev:* CTGTCCCTCGGCTTTGACAT	83

ATP2A2, sarcoendoplasmic reticulum Ca^2+^-ATPase 2; COL1A1, collagen type I alpha 1 chain; COL3A1, collagen type III alpha 1 chain; EDN1, endothelin 1; GAPDH, glyceraldehyde-3-phosphate dehydrogenase; MYH6, myosin heavy chain 6; MYH7, myosin heavy chain 7; NPPB, natriuretic peptide B; RPL4, ribosomal protein L4; RyR2, ryanodine receptor 2; TNF, tumor necrosis factor-α.

### 2.7 Western blot

Myocardial phospholamban (PLB), its phosphorylated form (p-PLB), and sarcoendoplasmic reticulum Ca^2+^-ATPase (SERCA) expression were assessed by western blot. RV samples were homogenized in modified RIPA buffer (50 mM Tris-HCl pH 8.0, 150 mM NaCl, 1% NP-40, 0.5% sodium deoxycholate and 0.1%–0.5% SDS) supplemented with protease (Sigma P8340) and phosphatase (Sigma P0044, Sigma P5726) inhibitors (1:100) using 1.4 mm zirconium oxide beads (Precelleys, Bertin Technologies) and tissue homogenizer (Minilys, Bertin Technologies). Total protein concentration was quantified using bovine serum albumin (BSA) as a standard (Bradford assay, Bio-Rad) and the sample concentration was adjusted accordingly. A volume of Laemmli Buffer 4× (8% SDS, 20% glycerol, 0.004% bromophenol blue and 100 mM Tris-HCl, pH 7.4) and DTT 10× (1.25 M) was added to the protein lysate and the solution was heated for 5 min at 95°C. 20 μg of RV protein were separated by electrophoresis using a 4%–20% gradient polyacrylamide gel (Criterion™ TGX™ Precast Gels, Bio-Rad) and then electroblotted onto nitrocellulose membranes (Bio-Rad). Blots were blocked for 1 h with 5% bovine serum albumin in Tris-buffered saline with Tween 20 (TBS-T; 50 mM Tris-HCl, 150 mM NaCl, pH 7.4, 0.1% Tween 20) for PLB and p-PLB detection and with 5% non-fat dry milk in TBS-T for SERCA analysis. PLB and p-PLB blots were transferred to 0.1% sodium azide solution (1:250 dilution) and primary antibodies to PLB (MA3-922, Invitrogen) and p-PLB (#8496, Cell Signaling) were added whereas blots for SERCA analysis were transferred to 5% non-fat dry milk in TBS-T (1:250 dilution) with primary antibodies to SERCA (ab3625, Abcam) and GAPDH (ab8245, Abcam). After overnight incubation at 4°C, blots were subsequently incubated in 0.5% non-fat dry milk in TBS-T with 700 nm and 800 nm infra-red dye-conjugated antibodies (IRDye® 680LT and 800W, LI-COR) for 60 min at room temperature and imaged by scanning at 800 and 700 nm with the Odyssey Infrared Imaging System (LICOR Biosciences). Experiments were carried out in duplicate. No differences were observed in GAPDH levels between groups. Data are presented relative to Sham levels.

### 2.8 Enzyme-linked immunosorbent assays

Previously aliquoted plasma samples were thawed on ice and assayed for N-terminal pro–B-type natriuretic peptide (NT-proBNP; CSB-EQ027465PI, Cusabio) and endothelin-1 (ET-1; ADI-900-020A, Enzo Life Sciences Inc) in duplicate according to the manufacturer's instructions.

### 2.9 Statistical analysis

Tabular data and graphs show the mean ± standard error of the mean for normally distributed data or the median and range and box plots otherwise, respectively. For normally distributed data, groups were compared by Student's or Welch *t*-test in case of homogeneous or non-homogeneous variance, respectively. The Mann–Whitney *U*-test was employed whenever deviation from normality was observed according to the Shapiro–Wilk test. Analysis of covariance was employed for data derived from linear or exponential fitting in PV relationships, where data were previously log-transformed whenever necessary to meet assumptions. Mixed Linear models with fixed effect for group and random intercepts by animal plus a variance component for each myocyte were performed for skinned RV cardiomyocytes studies, according to Sikkel et al. (22). Significance was set at 2-tailed *P* < 0.05. Statistical analysis and figures were performed with Python (key packages: numpy, pandas, statsmodels, scipy, and matplotlib).

## 3 Results

### 3.1 Repeated long suture PA embolization induces CTEPH

Repeated weekly embolization of the PA using long silk sutures resulted in a progressive and sustained elevation of mPAP, which was generally well tolerated by most animals (Figure 1D). Despite stringent criteria for discontinuing embolization, two CTEPH animals died during model induction. One animal succumbed to acute RV failure refractory to inotropic support during the third embolization (mPAP > 60 mmHg with mBP < 35 mmHg). The second animal was found dead the day after the second intervention, with acute RV failure considered the most likely cause.

At each embolization, major branches of the pulmonary arteries were occluded, and the obstruction persisted in follow-up. At necropsy, suture plugs accompanied by fibrin deposition were observed within large pulmonary arteries of CTEPH, but the right cranial lobe artery was unobstructed (Figures 1C,E). A cephalad shift in pulmonary perfusion was also observed and can be clearly appraised in the final angiography in Figure 1C. Supplementary Video 2 shows full PA angiograms.

On terminal evaluation, mPAP was substantially higher in CTEPH compared with Sham (51 ± 3 vs. 20 ± 1 mmHg, respectively; *P* < 0.001), there was also a clear reduction of CO (4.2 ± 0.2 vs. 6.6 ± 0.9 L.min^−1^; *P* = 0.042) yielding remarkably elevated pulmonary vascular resistance (PVR; 10.0 ± 1.1 vs. 1.5 ± 0.5 WU; *P* < 0.001). Of note, since wedged pressure could not be obtained reliably, we employed LV end-diastolic pressure (EDP) from PV loops in the estimation of PVR. CTEPH was induced consistently across animals (Figure 1D). CTEPH also showed a trend towards decreased weight gain on follow-up (Table 2).

**Table 2:** Morphometrics, echocardiography and hemodynamic evaluation.

	Sham (*n* = 6)	CTEPH (*n* = 7)	*P* value
Morphometrics			
BW*, kg*	62 ± 2	54 ± 4	0.172
RV/BW	1.1 ± 0.0	1.6 ± 0.2	**0**.**031**
Echocardiography			
PAAT/PAET	0.48 ± 0.03	0.35 ± 0.03	**0**.**005**
RVWT, mm	5 ± 0	8 ± 1	**0**.**023**
EI	0.95 (0.91–1.13)	1.19 (1.05–1.70)	**0**.**005**
TAPSE, mm	24 ± 3	15 ± 1	**0**.**009**
Right heart catheterization			
HR, bpm	103 ± 6	104 ± 8	0.896
mBP, mmHg	95 ± 6	83 ± 9	0.319
PAP (s/m/d), mmHg	32 ± 2/24 ± 1/14 ± 2	70 ± 4/51 ± 3/38 ± 3	**<0**.**001**
CO, L.min^−1^	6.6 ± 0.9	4.2 ± 0.2	**0**.**042**
PVR, WU	1.5 ± 0.5	10.0 ± 1.1	**<0**.**001**
Pressure-volume data			
Right ventricle			
P_max_, mmHg	34 ± 1	63 ± 4	**<0**.**001**
EF, %	51 ± 3	35 ± 2	**<0**.**001**
dP/dt_max_, mmHg.s^−1^	255 ± 25	348 ± 39	0.082
PRSW, mmHg	13 ± 4	27 ± 3	0.009
E_a_, mmHg.mL^−1^	0.5 ± 0.0	1.2 ± 0.2	**0**.**012**
E_es_, mmHg.mL^−1^	0.2 (0.1–1.1)	2.0 (0.6–5.3)	**0**.**005**
VVC	0.94 ± 0.38	2.1 ± 0.4	0.056
EDP, mmHg	8 ± 1	12 ± 1	**0**.**036**
EDV, mL	115 ± 21	125 ± 10	0.295
τ, ms	13 ± 3	20 ± 2	**0**.**041**
β, mmHg.mL^−1^	0.015 (0.001–0.042)	0.038 (0.021–0.176)	**0**.**031**
Left ventricle			
Pmax, mmHg	108 (90–129)	104 (86–108)	0.485
EF, %	64 ± 4	62 ± 2	0.241
dP/dt_max_, mmHg.s^−1^	788 ± 48	872 ± 143	0.590
PRSW, mmHg	100 ± 22	66 ± 5	0.309
EDP, mmHg	14 (11–24)	11 (9–19)	0.065
EDV, mL	110 ± 15	86 ± 15	0.286
τ, ms	15 ± 2	19 ± 1	0.128
β, mmHg. mL^−1^	0.036 ± 0.001	0.063 ± 0.001	0.116

BW, body weight; CO, cardiac output; dP/dt_max_, peak rate of pressure rise; E_a_, pulmonary arterial elastance; EI, eccentricity index; EDV, end-diastolic volume; EDP, end-diastolic pressure; E_es_, end-systolic elastance; EF, ejection fraction; HR, heart rate; mBP, mean systemic blood pressure; PAAT/PAET, ratio between pulmonary artery flow acceleration time and ejection time; PAP (s/m/d), pulmonary arterial pressure (systolic/mean/diastolic); P_max_, maximum developed pressure; PRSW, preload recruitable stroke work; PVR, pulmonary vascular resistance; RV, right ventricle; RVWT, right ventricular wall thickness; TAPSE, tricuspid annular plane systolic excursion; β, end-diastolic stiffness constant; *τ*, time-constant of isovolumic relaxation. Results are presented as mean ± standard error or median(range). Comparisons were performed using independent samples Student's or Welch's *t*-test for homogeneous and non-homogeneous variances, respectively, or Mann–Whitney *U*-test for non-normally distributed samples except for β and E_es_, data from PV relationships was analyzed with analysis of covariance, accounting for other fit parameters.

Bold values indicate p-values <0.05.

### 3.2 Severe CTEPH is accompanied by RV dysfunction

**Figure 2:**
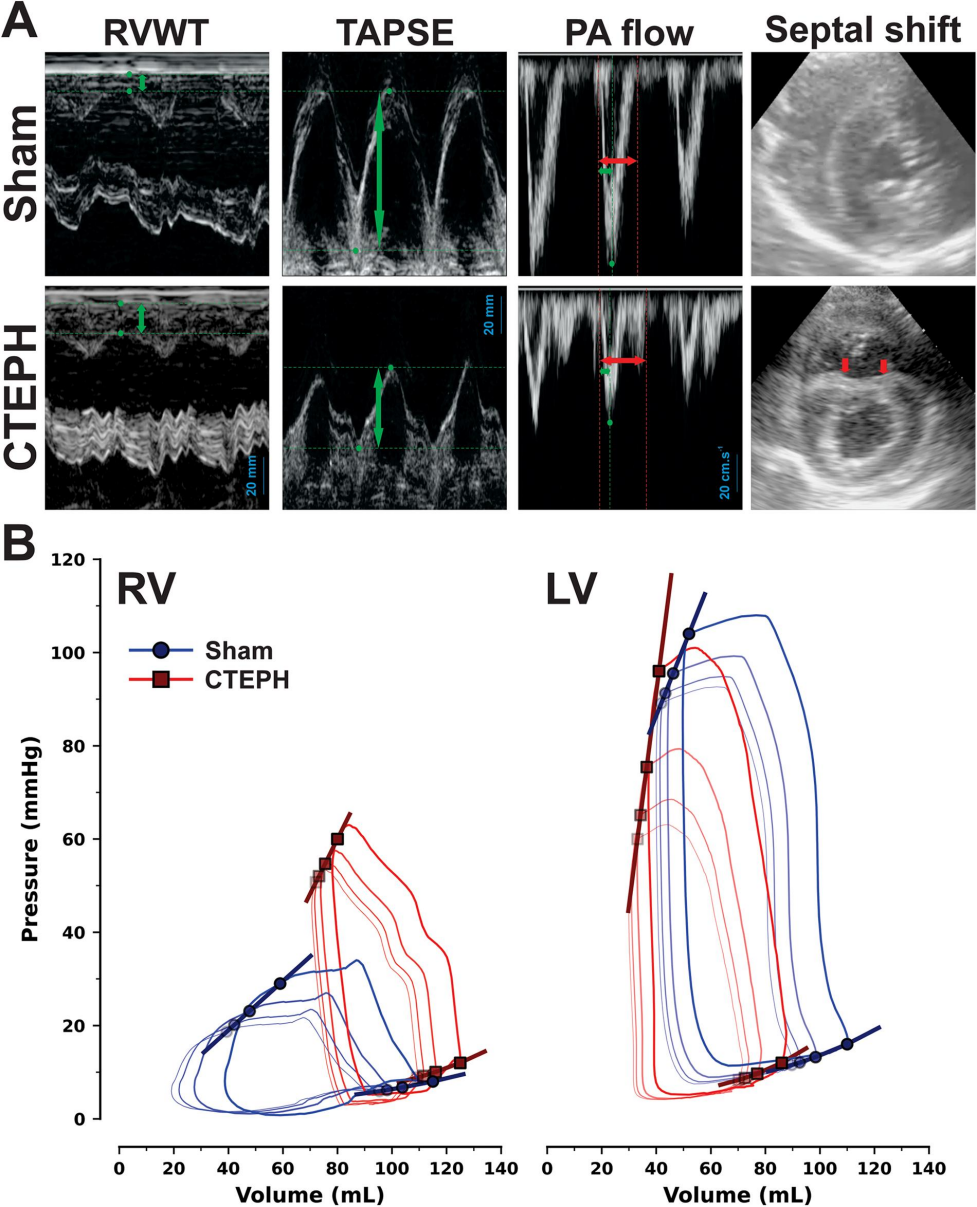
Representative echocardiography images **(A)** and right and left ventricular (RV and LV, respectively) pressure-volume loops in chronic thromboembolic pulmonary hypertension (CTEPH) and sham (sham). In **(A)**, acquisitions in M-mode illustrate measurements of RV wall thickness (RVWT) and tricuspid annular plane systolic excursion (TAPSE), pulsed-wave Doppler recording represents pulmonary artery (PA) flow and B-mode depicts transverse mid-ventricular right-to-left septal shift at early diastole in CTEPH (red arrows). In PA flow tracings, PA acceleration time (green arrows) as a fraction of ejection time (red arrows) is abbreviated in CTEPH compared with Sham. CTEPH also showed higher RVWT and lower TAPSE. In M-mode and pulsed-wave tracings time was normalized to represent 3 cycles. PV loops of inferior vena cava occlusions and corresponding end-systolic and end-diastolic PV relationships in **(B)** represent Sham (blue) and CTEPH (red) group averages, for further values and statistical analysis please consult Table 2.

On echocardiography, CTEPH presented lower TAPSE, increased RVWT as well as both short PA acceleration times and lengthened ejection times with late systolic notching in PA flow on pulsed-wave Doppler. Septal displacement towards the LV was particularly prominent in early diastole, leading to increased EI (Table 2 and representative tracings in Figure 2A). Supplementary Video 3 further illustrates septal shift and RV hypertrophy.

In PV hemodynamic analysis, the RV of CTEPH showed decreased CO, ejection fraction (35 ± 2 vs. 51 ± 3%; *P* < 0.001) and a trend towards dilation. Still, maximum pressure derivative (dP/dt_max_), preload recruitable stroke work (PRSW) and both pulmonary arterial elastance (E_a_) and RV end-systolic elastance (E_es_) were elevated, with preserved ventricle-vascular coupling (VVC), denoting overall stiffening of the right circulation in compensatory adaptation to PH. There was also delayed relaxation as assessed by time constant *τ* (20 ± 2 vs. 13 ± 3 ms; *P* *=* *0.041*), EDP elevation (12 ± 1 vs. 8 ± 1 mmHg; *P* = 0.036) and an upward shift in the EDPVR, as denoted by the rise in chamber stiffness constant β [0.038 [0.021−0.176] vs. 0.015 [0.001−0.042] mmHg.mL^−1^; *P* = 0.031]. No changes were observed in heart rate.

As for the LV, although systolic performance was unaltered there was a clear trend for unloading and impaired relaxation and filling, most likely due to ventricular interdependence. Averaged group PV loops, ESPVR and EDPVR from RV and LV IVC occlusions are depicted in Figure 2B and data is summarized in Table 2.

### 3.3 Structural, molecular and functional remodeling of the RV in CTEPH

**Figure 3:**
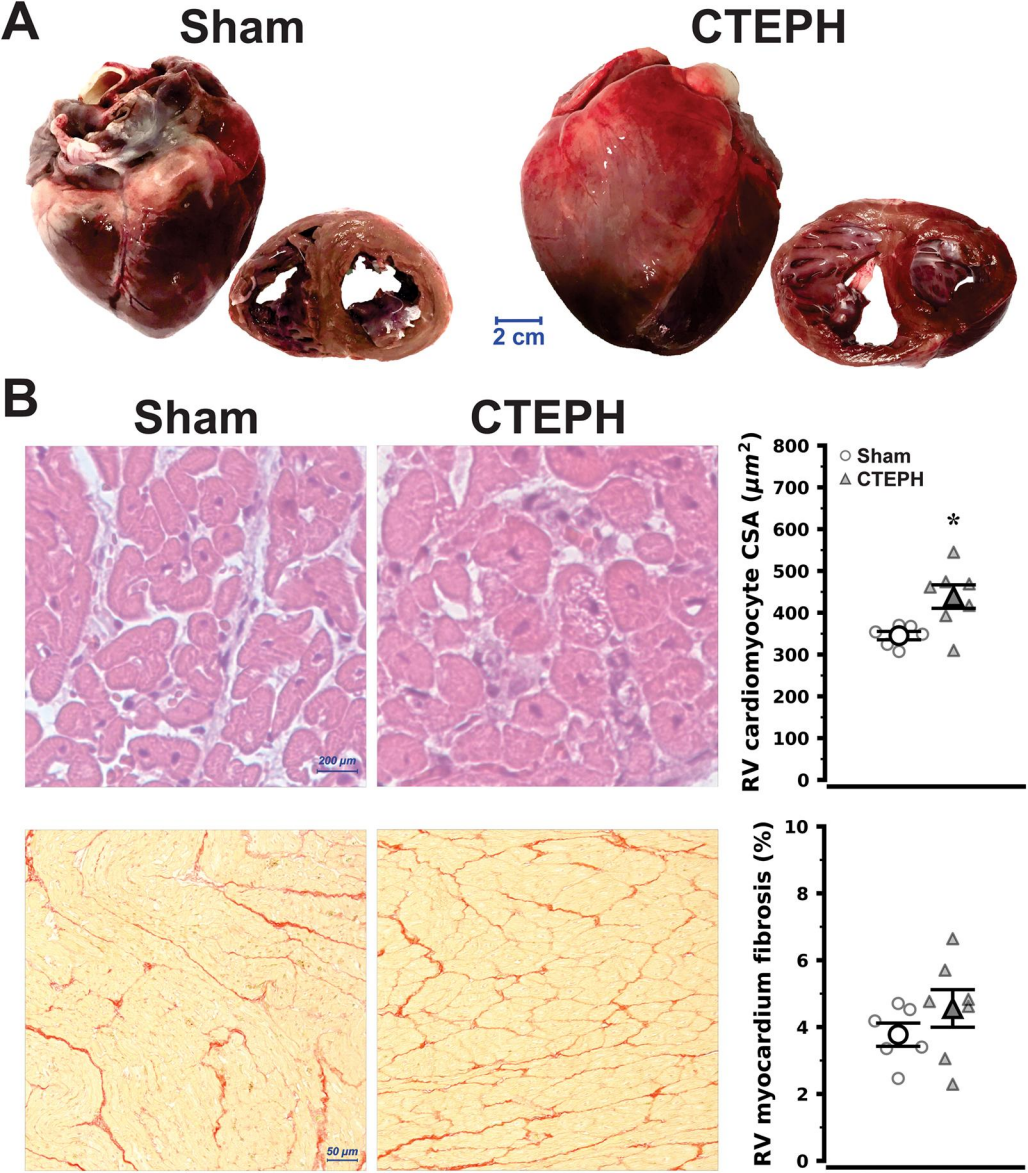
Cardiac morphology **(A)** and right ventricular (RV) histology in chronic thromboembolic pulmonary hypertension (CTEPH) and sham (sham). In **(A)**, we present side-by-side photographs of whole hearts and mid-ventricular cross-sections, where RV dilation and increased wall-thickness can be clearly appraised, values and statistical analysis are given in Table 2. In **(B)**, group representative RV myocardium hematoxylin and eosin (H&E) stained, assessing cardiomyocyte hypertrophy, and picrosirius red stained histological sections, assessing myocardial fibrosis (top and bottom, respectively), are presented alongside corresponding point plots with mean and error. CSA, cross-sectional area. *P = 0.013 vs. Sham by Student's *t*-test (*n* = 6 and 7 and Sham and CTEPH, respectively).

**Figure 4:**
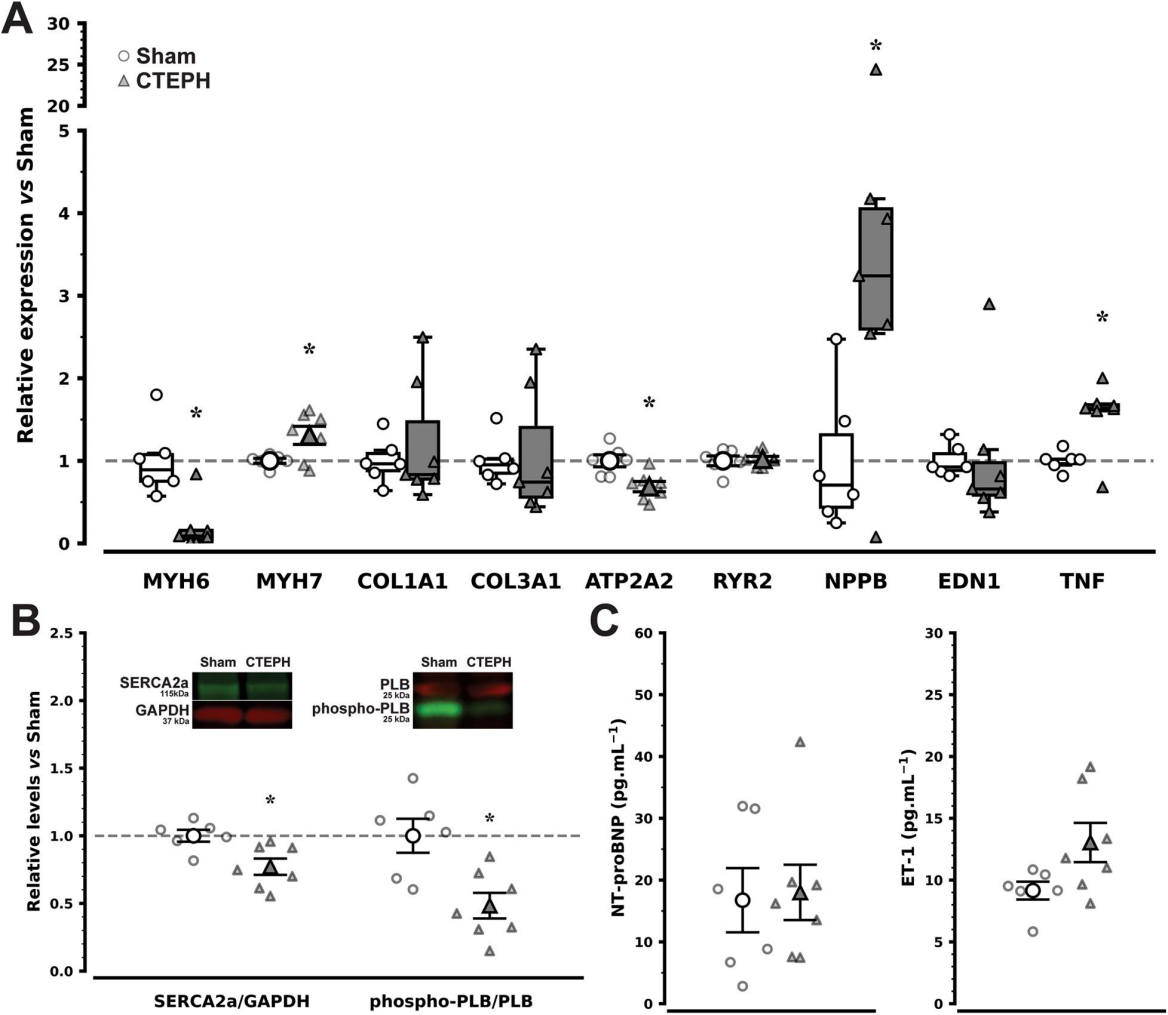
Right ventricular myocardium gene expression **(A)** and protein levels **(B)**, and circulating plasma mediator levels **(C)** in chronic thromboembolic pulmonary hypertension (CTEPH) and sham (sham). Gene expression data depicts genes involved in myofilament remodeling (MYH6 and MYH7, α- and β-myosin heavy chain isoforms, respectively), extracellular matrix fibrosis (COL1A1 and COL3A1, type I and III collagen chains, respectively), Ca^2+^-handling (ATP2A2, sarcoendoplasmic reticulum calcium ATPase 2; RYR2, cardiac ryanodine receptor), and neurohumoral mediators (NPPB, B-type natriuretic peptide; EDN1, endothelin-1; TNF, tumor necrosis factor-α). Data were averaged upon normalization for 2 internal control genes, GAPDH (glyceraldehyde-3-phosphate dehydrogenase) and RPL4 (ribosomal protein L4). Calcium-handling protein levels of sarcoendoplasmic reticulum Ca2+-ATPase 2a (SERCA2a) normalized for GAPDH and phospho-phospholamban (PLB) normalized for total PLB and representative Western blot bands are presented in **(B)**, whilst mediator plasma levels of N-terminal pro–B-type natriuretic peptide (NT-proBNP) and endothelin-1 (ET-1) are presented in panel C. In panels A and B data are presented relative to Sham reference levels (dashed lines). *P < 0.05 vs. Sham by Student's *t*-test or Mann–Whitney *U*-test, according to assumptions (*n* = 6 and 7 in Sham and CTEPH, respectively).

RV hypertrophy was evident at necropsy (Figure 3A), with increased RV weight in CTEPH (Table 2), as well as on histological sections from the RV myocardium, according to cardiomyocyte CSA (Figure 3B, top), nevertheless no significant differences in fibrosis percentage was noted (Figure 3B, bottom). These changes were paralleled by a shift towards cardiomyocyte fetal phenotype in gene expression, RV myocytes from CTEPH showed increased expression of MYH7, which codes for slow β-myosin heavy chain, and markedly reduced expression of fast α-myosin heavy chain encoded by MYH6, as well as raised expression of NPPB and TNF, which code B-type natriuretic peptide (BNP) and tumor necrosis factor-α, respectively. Collagen chain expression and RV myocardium gene expression of ET-1, however, were unchanged (Figure 4A). Moreover, despite RV overexpression of BNP no changes were observed in circulating plasma levels regarding N-terminal-proBNP whereas ET-1 plasma levels showed a trend for increase (Figure 4C; *P* = 0.059).

**Figure 5:**
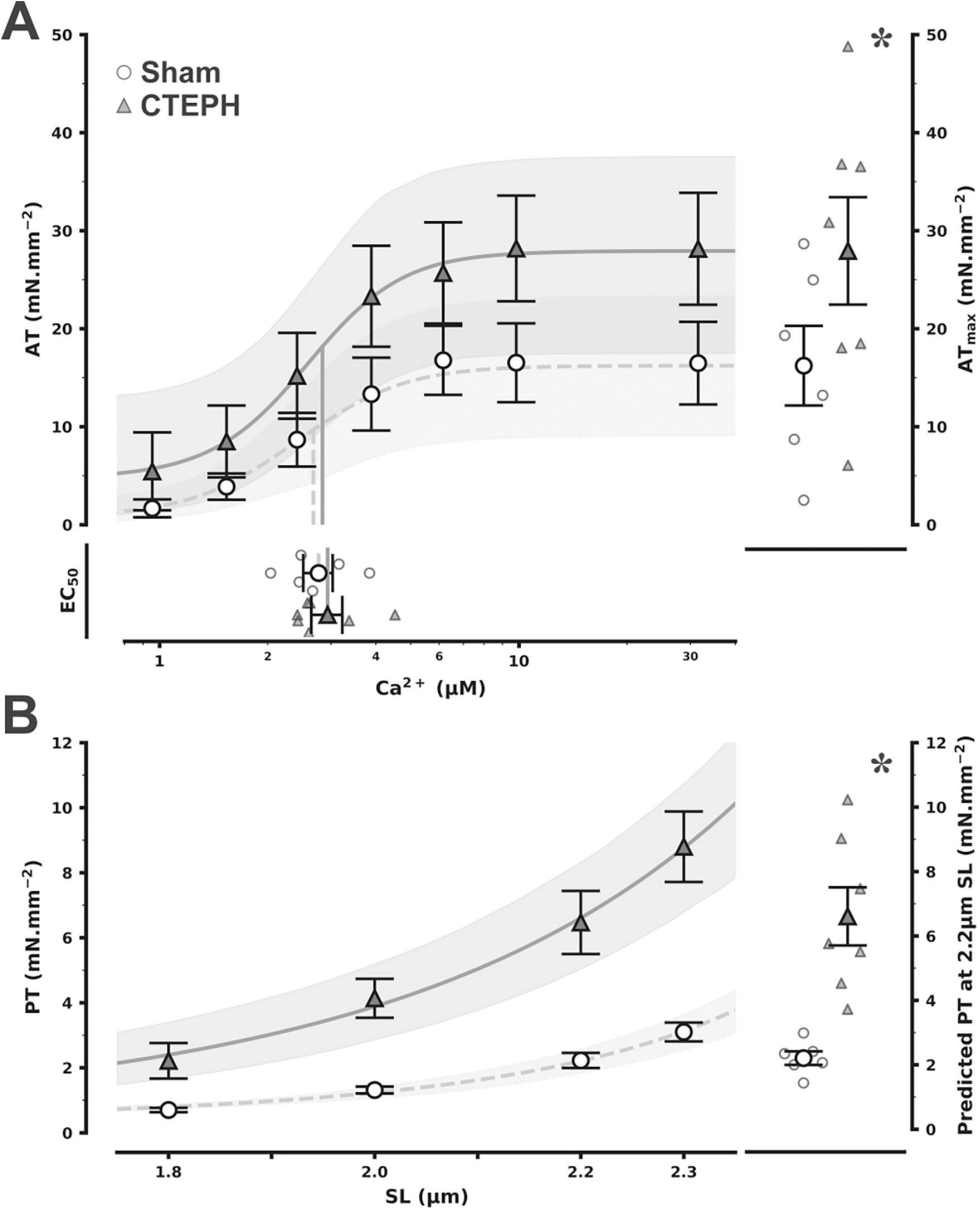
Calcium-active tension (AT) relationship **(A)** and sarcomere length (SL)-passive tension (PT) relationship in right ventricular cardiomyocytes from chronic thromboembolic pulmonary hypertension (CTEPH) and sham (sham) **(B)** main fits depict mean and error for the relationships along with 95% confidence intervals (obtained by bootstrapping with 5,000 resamples) for predicted fit lines according to the hill and exponential equations [**(A,B)**, respectively]. Based on fitting parameters, in **(A)**, drop lines represent half maximal effective Ca^2+^ concentration (EC_50_), a point plot with mean and error is given below while a corresponding subplot with maximum AT (AT_max_) is represented on the right. Likewise, a point plot with mean and error is shown on the right for PT at 2.2 µm SL in **(B)**. *P < 0.001 vs. Sham by mixed linear models (*n* = 6 and 7 and Sham and CTEPH, respectively, at least 5 myocytes per animal).

Skinned RV cardiomyocytes from CTEPH showed elevated Ca^2+^-active tension (AT) responses, with raised maximum AT but unchanged Ca^2+^ myofilament sensitivity as appraised by Ca^2+^ EC_50_ compared with Sham (Figure 5A), as well as increased passive stiffness as evidenced by the upward shift in SL-PT (Figure 5B). Calcium-handling proteins were also disturbed in CTEPH, SERCA2a protein levels (Figure 4B; full representative Western blot membranes are presented as Supplementary Figure 1 in Supplementary Material) and its ATP2A2 gene expression (Figure 4A) were both reduced along with hypophosphorylation of phospholamban (Figure 4B), both suggesting impaired Ca^2+^ reuptake. No changes were observed in the expression of the cardiac ryanodine receptor.

### 3.4 Distal pulmonary vasculopathy induced by CTEPH

**Figure 6:**
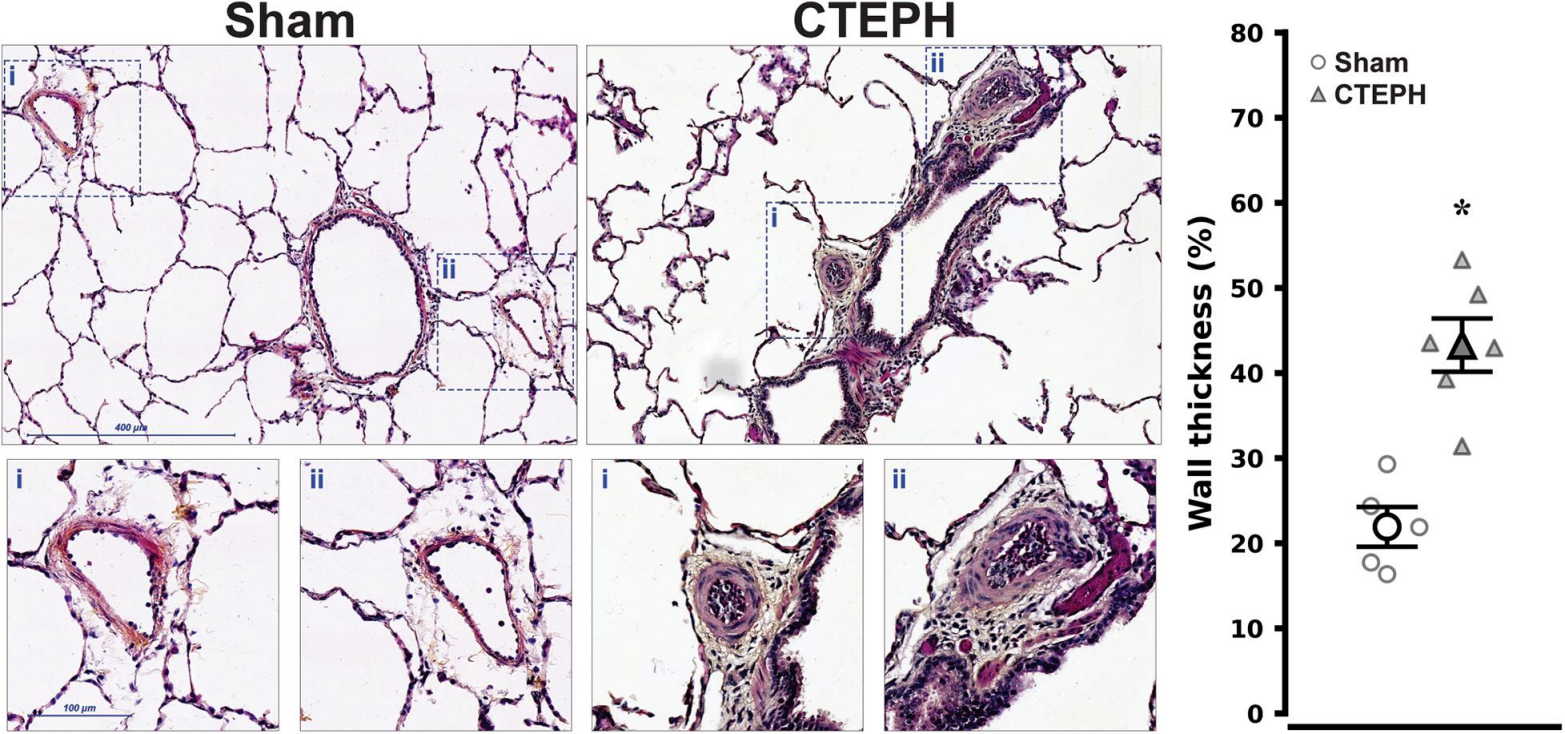
Lung arteriolar remodeling in chronic thromboembolic pulmonary hypertension (CTEPH) and sham (sham). Representative hematoxylin-eosin-saffron stained histological sections and individual arterioles insets from the right cranial lobe, which was preserved during the embolization process, are presented along with a corresponding point plot with mean and error showing percent wall thickness in arterioles with a diameter <100 µm. Medial hypertrophy is conspicuous in arterioles from CTEPH, denoting flow-induced distal vasculopathy. *P < 0.001 vs. Sham by Student's *t*-test (*n* = 5 and 6 in Sham and CTEPH, respectively).

Due to the pulmonary arteries anatomy of swine, the right cranial lobe artery was preserved. Indeed, a cephalad redirection of flow was clearly visible on angiograms. This overflow induced remodeling of distal pulmonary arterioles (<100 µm) in CTEPH lungs which showed increased media thickness compared with sham (43 ± 3 vs. 22 ± 2%, respectively; *P* < 0.001), as represented in histological sections from the right cranial lobe (Figure 6).

### 3.5 Pulmonary structural changes

**Figure 7:**
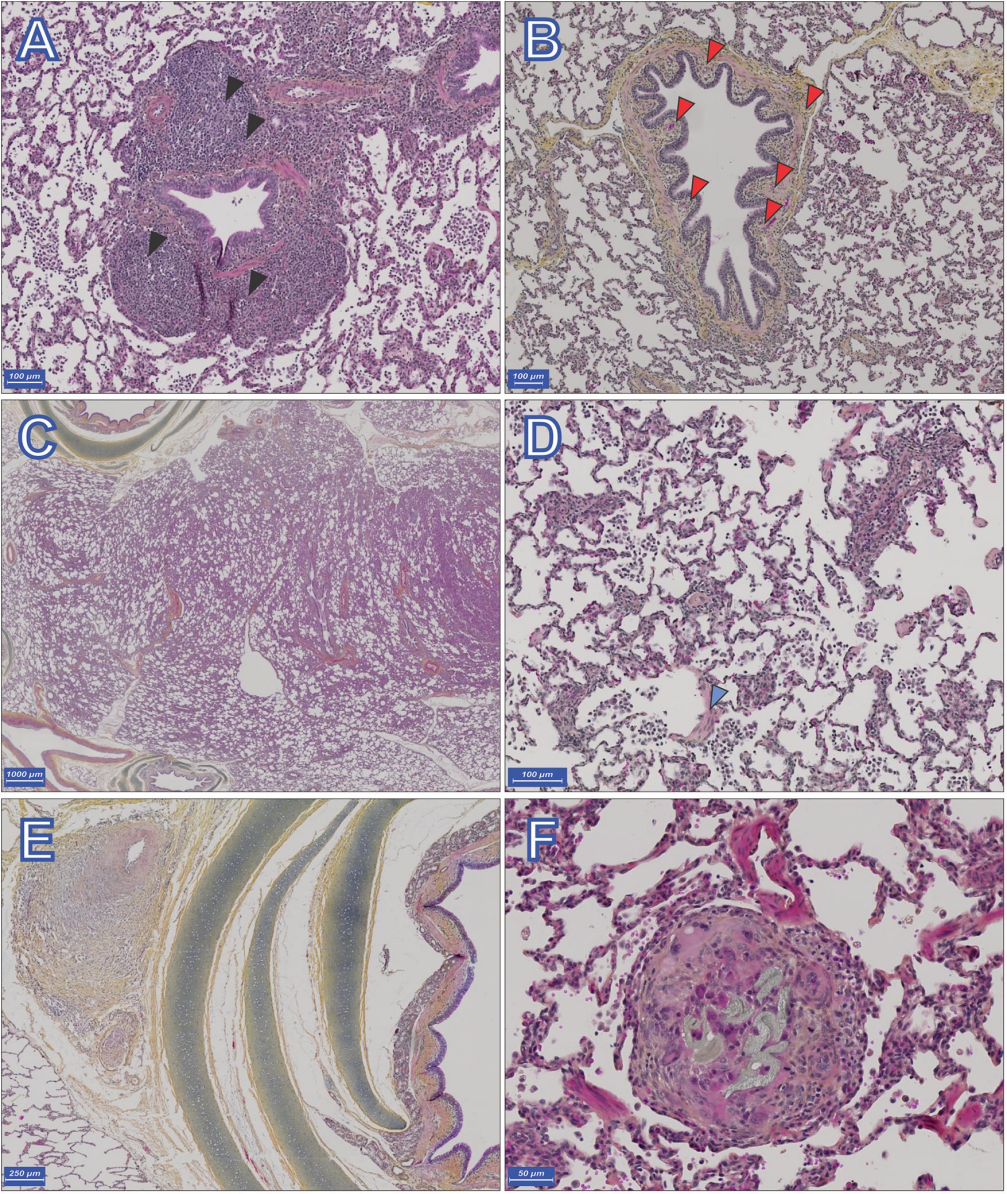
Representative histopathology of chronic thromboembolic pulmonary hypertension (CTEPH) lung sections stained with hematoxylin-eosin-saffron. Lungs displayed abundant bronchial follicular inflammation, with germinative center follicles [black arrow heads, **(A)**]. Pseudopolypoid hyperplasia of respiratory epithelium in bronchus, with prominent underlying bronchic vasculature [red arrow heads, **(B)**]. Distal and, more predominantly, proximal samples displaying Non Specific Interstitial Pneumopathy-pattern of alveolar wall thickening **(C)**. **(D)** shows abundant alveolar wall thickening as well as arteritis composed of mononucleated leucocytes, muscularized arterioles are in excessive number and show images of vasculitis, devoid of leucocytoclastic features. Macrophage overload is observed in alveoli, devoid of neutrophilic participation or pathogen identification. Note the presence of a de-epithelialized respiratory bronchiole (blue arrowhead). In **(E)** modification of bronchial arteries is identified in the bronchovascular bundle, with expansion of adventitia and media cellular recruitment of myofibroblasts and composition of a neomuscularised media. Only few leucocytes are found, in the periphery of the remodeling. **(F)** Giant cell reaction trying to resorb foreign body in a distal artery (silk thread). Scale bars are shown on the lower left corner of each panel.

CTEPH lungs displayed abundant peribronchovascular lymphoid inflammation, mainly characterized by follicular organization with germinative centers (Figure 7A), often associated with multiple inflammatory cell types, including neutrophils, in both central and distal parts of the lung. The airways showed thickening of the mucosa, characterized by a pseudopolypoid appearance, sometimes sub-occlusive, raised by hyperplastic or aberrant bronchial vascularization (Figure 7B). Distant respiratory bronchioles were sometimes devoid of overlying epithelium, and extensive thickening of the alveolar wall was particularly noticeable in CTEPH lungs, while not very noticeable in Sham samples. These changes are morphologically like human Non-Specific Interstitial Pneumopathy (NSIP, Figure 7C), with a more cellular, or a mixed cellular and fibrous components in distal or proximal samples, respectively, suggesting a temporal and spatial gradient of CTEPH-related lesions (earlier lesions being more distant).

Pulmonary arteries showed vasculitis lesions without vascularitis or leukocytoclastic features, as well as intimal and medial hyperplasia with an increase in small distal muscularized vessels (Figure 7D), without significant intimal fibrosis or thickening. Aberrant or occlusive muscularization of the septal vessels was also observed. Bronchial vessels showed adventitial myxedema or centripetal remodeling of the media in CTEPH (Figure 7E). Although predominant in CTEPH, microthrombi were also found in Sham.

The obstructed lobes of CTEPH animals (basal portions) showed congestive and hemorrhagic changes in the bronchial tree and bronchial arteries similar to those described above. Follicular inflammation and expansion of the bronchial artery bed was evident (Supplementary Figure 2A,B in Supplementary Material), as well as pulmonary arterial medial hyperplasia (Supplementary Figure 2C in Supplementary Material). In addition, they were associated with an increase in the number of alveolar capillaries, which were also dilated, sometimes taking on a capillary hemangiomatosis-like appearance (Supplementary Figure 2D in Supplementary Material).

Inflammatory changes related to the intervention, such as intravascular foreign bodies, isolated or embedded in a giant cell resorptive inflammatory reaction, were observed in CTEPH (Figure 7F). No old nor organized thrombi or colander-like lesion were identified in the tissue samples taken from either group. No inflammatory changes of suspected septic origin, or tumoral features were identified.

On some of the sites of occlusion a fibrin and collagen cast is clearly identifiable around suture threads, with eccentric recanalization channels, a representative section is presented as Supplementary Figure 3 in Supplementary Material.

## 4 Discussion

In this work, we developed a severe and consistent CTEPH swine model characterized by severe PH, RV remodeling and dysfunction, and flow-induced distal pulmonary vasculopathy. To our knowledge, this represents the finest translational large animal model currently available for recapitulating key pathophysiological features of human CTEPH.

The development of effective therapies for PH has been hindered in part by the limited availability of animal models that faithfully reproduce the vascular remodeling and progressive RV failure observed in human disease (23). As outlined in the introduction, rodent models diverge substantially from human pathophysiology, contributing to persistent translational challenges. Although large animal models entail greater costs and logistical complexity, they offer superior physiological relevance and markedly enhance the potential for clinical translation (24).

The classical and still widely employed rat model of PH induced by monocrotaline (25, 26), is limited in swine, as it leads only to mild PH in minipigs (11). A single work shows significant PH development (mPAP of 55 mmHg at 12 weeks of follow-up) upon repeated administrations (27). In contrast, chronic hypoxia induces severe PH but is typically conducted in newborn animals and requires a significant investment in equipment (12). Regarding CTEPH, embolization of autologous thrombi would be the preferred approach because it entirely mimics disease pathophysiology. Nevertheless, this approach has failed, even when endogenous fibrinolysis was inhibited (28). This is out of scope of the current work, but large animal models of CTEPH have been thoroughly reviewed elsewhere (9). To summarize, development of large animal CTEPH models is challenging (16), currently available swine models are complex and time consuming, requiring multiple hits, such as surgical ligation of the left PA (13), a combination of proximal and distal embolization (14), or long-term successive bead embolization with (15, 16, 29) or without additional pharmacological interventions (17, 30, 31). Moreover, they usually lead to mild phenotypes (15), thus the maximum reported mPAP of 39 mmHg (9) is quite lower than average values for mPAP of 46 mmHg in humans (18).

Because of its simplicity, in a pilot study, we attempted to replicate the model developed by Aguero et al. (14). In this pilot study some animals died due to acute RV failure during the embolization process, and the final rise in PA pressure was mild. Microsphere embolization was mostly targeting very small peripheral arteries, and short (1.5 cm) sutures were also not clogging large arteries (a necropsy image is shown in Supplementary Figure 4 of Supplementary Material) which is at marked contrast to clinical findings (32). We therefore hypothesized that simple embolization of larger length sutures would cause substantial obstruction of larger arteries better mimicking CTEPH's pathophysiology.

We designed the study protocol taking into consideration that cautious repeat embolization would be warranted. Swine grows very quickly to weights that render animal handling unfeasible, thus waiting for disease progression after a single embolization seemed unattainable. On the other hand, extensive PA obstruction could easily lead to overt RV failure and animal loss, therefore small prudent embolization steps were deemed crucial to progressive but timely model development. We systematically started by the smaller left PA vascular territory, with a low threshold for intervention interruption based on PA pressure rise, CO or systemic BP decline, or simply because the angiography goal of vessel obstruction had been achieved. All interventions were carried out on room air ventilation and normocapnia so the impact of embolization on hemodynamics was not misjudged. To allow RV adaptation to impending afterload we repeated interventions weekly targeting the right PA territory while sparing the right cranial lobe artery in a second intervention and again the left PA in a third intervention. In these subsequent interventions PA pressure was allowed to rise to higher levels. We believe this was crucial to minimize animal losses to follow up while still achieving a severe PH phenotype.

Silk sutures are widely available at a low cost compared to commercial coils and clearly accomplish the goal of obstructing large pulmonary arteries if long sutures are used. Suture embolization led to a significant obstruction of pulmonary vascular blood flow, in both branches of the PA which persisted 4 weeks after the last embolization step. Occluding suture clots were observed alongside fibrin clusters in both pulmonary arteries and their main branches, except for the right cranial lobe artery, inducing a sustained chronic elevation of mPAP, averaging 50 mmHg, quite similar to values reported in humans with severe PH (18) and at clear contrast with most currently published large animal models (14, 15, 24, 29).

In CTEPH's pathophysiology persistent obstruction of large and/or middle-sized pulmonary arteries by thrombi is followed by small-vessel abnormalities and microvascular remodeling that further contribute to PVR rise, posing a strong impact on clinical outcomes (32). Remodeling occurs distally to obstructed sites possibly due to collateral flow from bronchial arteries but mostly in unobstructed arterial territories as a direct result of flow redirection. High flow-related shear stress (33) leads to notorious remodeling of small arteries that typically display medial hypertrophy. This is well documented in CTEPH patients (34, 35) and swine models (13–15), and is also a remarkable feature of the current model. Recent evidence suggests a potential role for ET-1 in smooth muscle cell proliferation that accompanies small-vessel disease in CTEPH (36), however, in the current work we only observed a non-significant trend for increased ET-1 plasma levels.

On the pathologic point of view, deep vascular changes in most segments are consistent with previous animal (37) and human (35) studies, despite a lack of colander-like lesions or deep organized thrombi, which might be related to sampling bias, as CTEPH lesions can be heterogeneous in time and space, and even highly focal. The several modifications in the bronchial circulation also support the pathobiological validity of the model (38, 39).

The peribronchovascular changes and follicular expansion observed in the cranial lobes most likely correspond to the formation of tertiary lymphoid tissues (tLTs), as seen in other forms of PH and interstitial lung diseases (ILD, CTD-ILD) (40, 41). These tLTs represent a local and organized immune response combining T- and B-cell areas, dendritic cells, and high endothelial venules, indicating persistent immune activation within the lung parenchyma, contributing to self-sustaining inflammation and chronic vascular remodeling (40, 42). In obstructed areas, focal ischemia followed by reperfusion, due to significant bronchopulmonary collateral blood flow (43) might also mediate the observed inflammatory response.

Inflammation is now recognized as a major determinant of pulmonary vascular remodeling (44). Perivascular infiltration by macrophages, lymphocytes, and dendritic cells (45) often precedes medial hypertrophy, underlined by a Th17/IL-17 axis activation fueling fibroblast activation and smooth muscle cell proliferation (42, 46, 47).

Extrapulmonary inflammatory signals may also amplify local pulmonary responses. In advanced PH, hepatic congestion and portal hypertension can impair intestinal barrier integrity, leading to bacterial translocation and mild endotoxemia (48), through systemic release of lipopolysaccharides that through a TLR4/CD14 culminate in overproduction of TNF- α and IL-6 sustaining a pro-inflammatory (49).

The coexistence of organized lymphoid aggregates, NSIP-like lesions, and inflammatory arteriolitis in our model suggest a continuum between inflammation and remodeling, possibly maintained by mechanical stimuli. Experimental studies have shown that abnormal blood-flow conditions (high or oscillatory shear stress) promote endothelial activation, oxidative stress, and immune-cell recruitment (50). This inflammation-oxidation-shear coupling may explain the simultaneous presence, in our model, of chronic embolization, vasculitis, and lymphoid inflammation.

Altogether, the inflammatory response observed in cranial lobes may represent an adaptive process secondary to blood-flow redistribution and sustained mechanical and oxidative stress. Chronic inflammation supported by tLTs could drive the transition from granulomatous reaction to persistent vascular and interstitial remodeling. The presence of fibrin microthrombi, venous edema, and vasculitis supports the existence of a pro-inflammatory and pro-thrombotic microenvironment, similar to that observed in early-stage CTEPH, and are consistent with those observed in other CTEPH models.

This new CTEPH experimental model also entails substantial RV remodeling and dysfunction as well as some degree of LV unloading, which are relevant features to clinical translation in CTEPH research. The thin-walled RV is ill-prepared to face increased afterload and adapts simultaneously with hypertrophy, stiffening, and dilation. It enhances its contractility attempting to preserve CO by preload reserve recruitment through the Frank-Starling mechanism (51). The extent of dilation and ejection fraction decay seem to be worse with proximal PA obstruction (52). This CTEPH model consistently replicates most of the echocardiography and hemodynamic features of human CTEPH. Nonetheless, it must be pointed out that CO was only mildly impaired in CTEPH swine, there was considerable variability in the extent of dilation and only a trend for higher RV EDV. The rise in E_es_ was still able to offset the increase in PA E_a_, thus preserving VVC which is not what would be expected in patients with longstanding CTEPH (53). We interpret these findings considering both the limited time course of evolution of our model, only 4 weeks after the last embolization and 6 weeks from the start of the experimental protocol, as well as the age of the animals. As in congenital heart disease and pediatric cardiology, where children tolerate RV afterload elevation remarkably well, young animals typically tolerate RV afterload better (54). Indeed, the same reasons might explain why no changes were observed in RV fibrosis or circulating levels of NT-proBNP, which would be expected in clinical CTEPH (32). We foresee that a more prolonged follow-up would have led to declining VVC, further deterioration of CO and overall health status, as well as RV fibrosis.

Still, we observed substantial adaptations at the cardiomyocyte level, namely a shift in myosin heavy chain expression favoring the slower and more energetically efficient β-chain which is better suited to withstand overload and therefore, may be viewed as adaptive, but also decreased expression of SERCA2a and hypophosphorylation of phospholamban, both favoring impaired Ca^2+^-reuptake by the sarcoendoplasmic reticulum. The latter are clearly maladaptive and may underlie both delayed relaxation and increased filling pressures due to residual crossbridge formation, the hallmarks of diastolic dysfunction. They also suggest a clear progression towards systolic dysfunction and a decline in RV contractile reserve, due to sarcoendoplasmic reticulum Ca^2+^ store depletion. Similar changes in Ca^2+^-handling proteins were previously reported in PAH patients and other CTEPH models (55). Indeed, despite the absence of fibrosis we documented diastolic stiffening which is clearly associated with disease progression and survival in PH (56), and also in CTEPH patients (57) and PH models preceding overt RV systolic dysfunction (26). Beyond cytosolic Ca^2+^ overload and residual crossbridge formation, intrinsic RV cardiomyocyte stiffening as observed in the upward shift in the SL-PT relationships suggests that myofilament disturbances, namely in titin, might be involved even before fibrosis sets in, a subject that requires further research.

As for the LV, while systolic function is usually preserved, diastolic dysfunction is common in CTEPH (58) due to both parallel (septal bulging) and series (underfilling) ventricular interaction or interdependence. Although these changes were not significant, we observed strong trends for lower LV EDV and EDP in LV PV loops, as well as increased eccentricity index at early diastole on echocardiography. Both decreased CO due to series interdependence, and delayed RV relaxation at higher pressures which dictates rapid leftward septal shift at early diastole and delayed LV filling by parallel interdependence (59) may have contributed to LV unloading (Supplementary Figure 5 in Supplementary Material illustrates this).

## 5 Limitations

This work has several limitations that must be addressed. Our goal was to develop a practical and cost-effective CTEPH animal model suitable for preclinical translational research. To this end, we restricted our proof-of-concept sample to young, uncastrated male swine from a readily available strain. Consequently, our findings may not be generalizable to females, older animals, or other swine strains. Moreover, though easily available and inexpensive, silk sutures do not entirely mimic chronic emboli in human CTEPH. As previously stated, this CTEPH model shows preserved VVC and only mildly impaired CO. We believe that increasing the follow-up period might have yielded a severer phenotype, but this would raise concerns regarding animal weight and well-being. The lack of resolution and digital subtraction imaging capabilities of our angiography device prevented us from having a clear picture of the exact segments obstructed. We did not plan in advance to quantify titin isoform or phosphorylation status, conducting Ca^2+^ transient studies in RV cardiomyocytes (which was performed in mid-wall transmural samples, not taking into account different RV anatomical locations or wall layers) or examining endothelial dysfunction in lung artery endothelium, which would strengthen the description of the CTEPH phenotype and complement available information. We failed to collect lung samples for molecular biology studies and only employed basic histological analyses, which hinders our characterization of the cellular and molecular pathways involved in the development of the observed distal and peribronchial inflammation, as well as the nature of the lymphoid aggregates Furthermore, extrapulmonary inflammation (due to hepatic congestion and altered intestinal permeability leading to endotoxemia) cannot be ruled out as a cause of the observed inflammatory changes, and due to the lack of morphometric and or biochemical analysis of liver function we are unable to appreciate this. Lastly, few tissular alterations were seen in samples from the Sham group, including NISP-like pulmonary arterial leukocyte margination, which are likely associated with hemodynamic microdisturbances caused by repeated right heart catheterization even in the absence of foreign body injections. The florid follicular lymphoid reaction could not be studied in depth but remains an interesting field for further use of the model. No leukocytoclasia or pathogen pooling allowed us to exclude an intravascular septic cause, which could bias our interpretations.

## 6 Conclusions

We developed a new, consistent and cost-effective animal model of CTEPH by injecting long sutures into the pulmonary circulation, without the need for additional pharmacological or interventional hits. These findings strongly suggest that this swine model of CTEPH effectively reproduces the features of human CTEPH, including RV structural and functional remodeling. While it is not ideal to study overt RV failure since CO is only slightly decreased, the current phenotype is adequate for preclinical testing and constitutes a starting point for future longitudinal studies.

## Data Availability

The original contributions presented in the study are included in the article/Supplementary Material, further inquiries can be directed to the corresponding author.
