# Supplementary material for: A swine model of severe chronic thromboembolic pulmonary hypertension induced by repeated pulmonary artery long suture injection
Source: Front Cardiovasc Med. 2026 Jan 16;12:1736958. doi: 10.3389/fcvm.2025.1736958 (PMC12856298; doi:10.3389/fcvm.2025.1736958)
Supplement: Supplementary file 4 [file Datasheet1.pdf]

Supplementary Material

Supplementary Figures

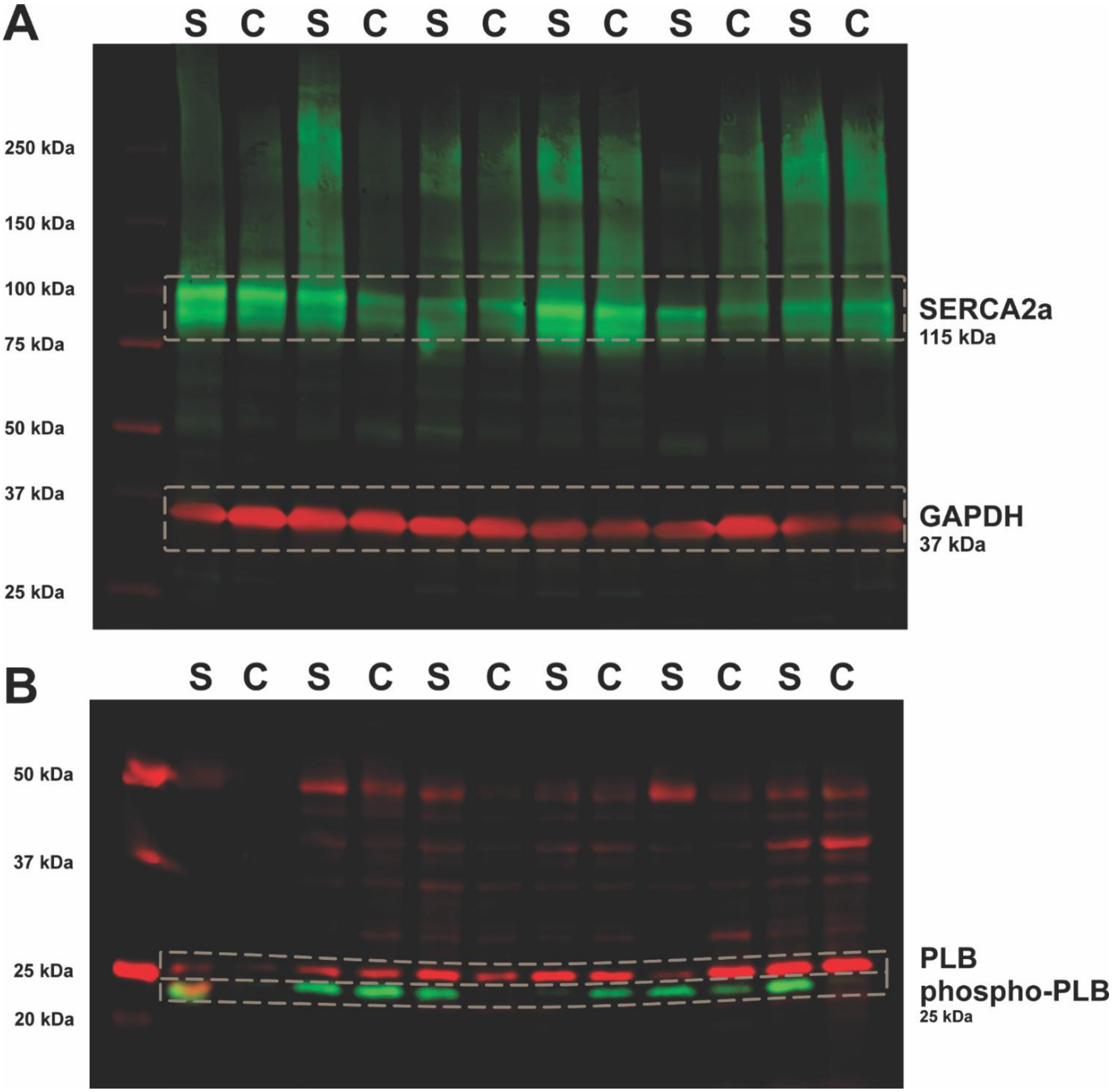

Supplementary Figure 1. Representative Western blot membranes. Sarcoendoplasmic reticulum Ca2+-ATPase 2a (SERCA2a, green) and control protein glyceraldehyde-3-phosphate dehydrogenase (GAPDH, red) levels (Panel A), and phospholamban (PLB, red) and phospho-PLB (green) levels (Panel B) assayed by LI-COR Odyssey imaging system in representative full Western blot membranes. Right ventricular samples from Sham (S) and chronic thromboembolic pulmonary hypertension

(CTEPH, C) are distributed in alternate lanes. The leftmost lane holds the protein molecular weight marker. Simultaneous acquisitions on both the green and right channels (800 and 700nm, respectively) are presented for easier visual comparison though the signal may be saturated in some bands (actual analysis took place in acquisitions optimized in each of the channels).

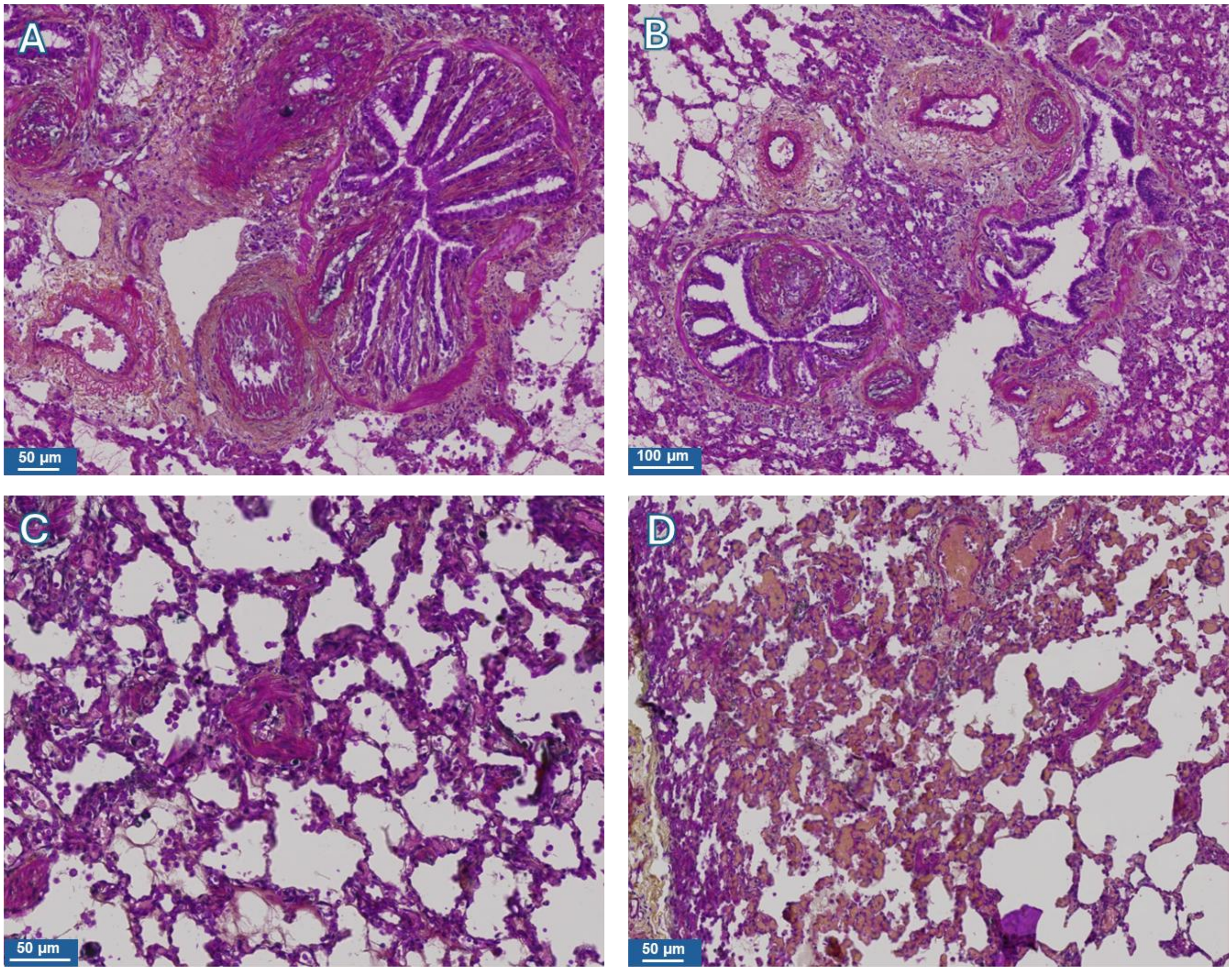

Supplementary Figure 3. Illustrative histology section of the basal obstructed lobes. CTEPH obstructed lungs showed pseudo papillary hyperplasia of respiratory mucosa, follicular inflammation and expansion of the bronchial arterial bed (Panels A and B). Pulmonary artery media hyperplasia (Panel C) and capillary hemangiomatosis-like congestion (Panel D) were also observed.

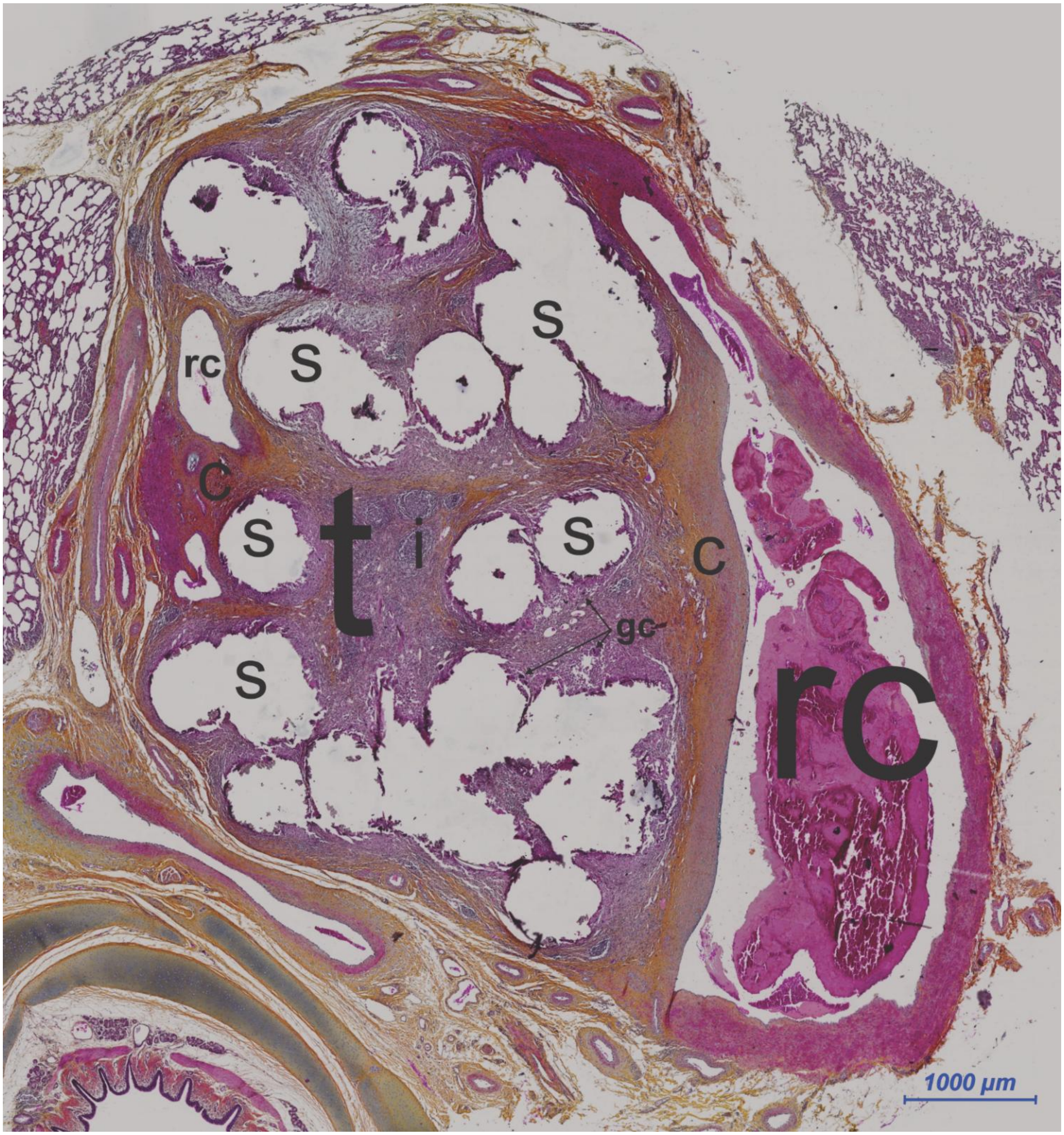

Supplementary Figure 3. Illustrative histology section of an obstructed pulmonary artery branch. Pulmonary artery branch showing luminal occlusion by organized thrombotic material (t), composed of silk material (s) surrounded by a mild foreign body inflammatory (i) and giant cell reaction (gc) along with dense collagenous tissue and interspersed fibroblasts (c). Eccentric recanalization with residual thrombus is also visible (rc). hematoxylin-eosin-saffron staining.

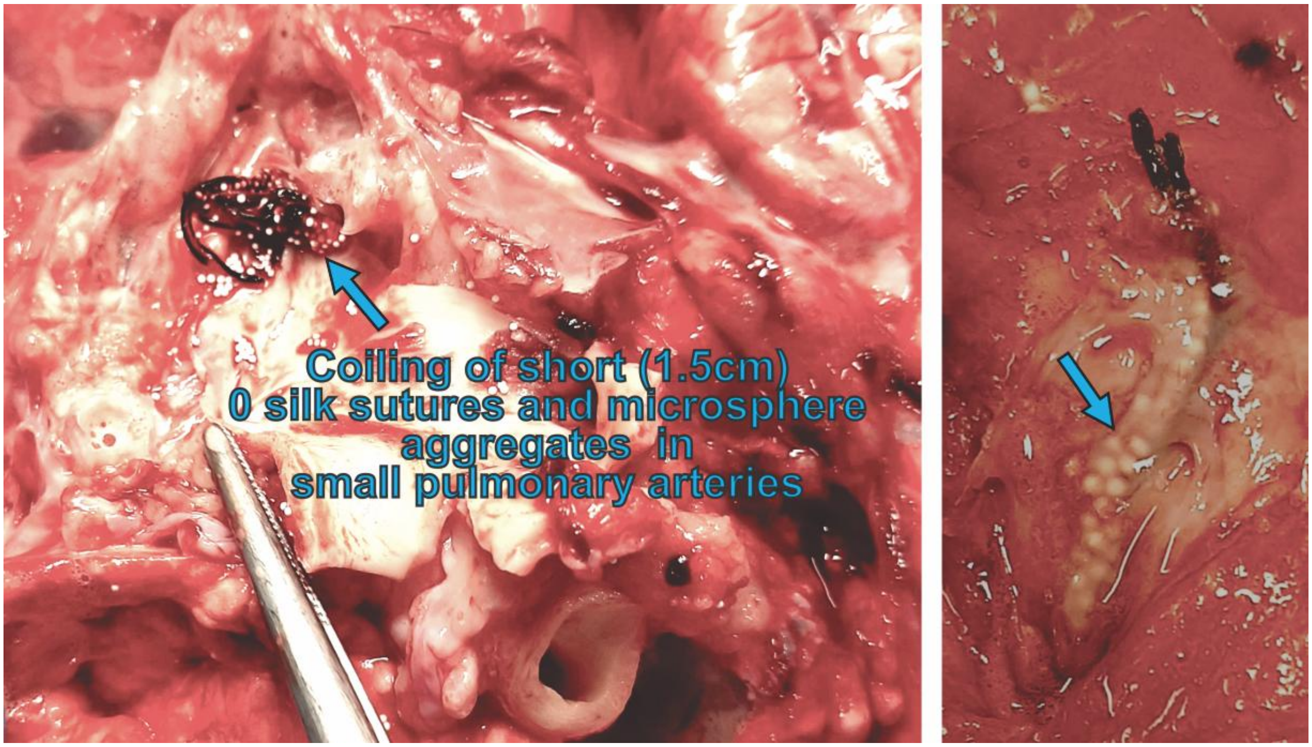

Supplementary Figure 4. Necropsy findings in pilot study reproducing the model developed by Aguero et al. (reference 14 from the main manuscript). On necropsy, obstructions comprising suture and microspheres were found mainly in distal small sized pulmonary arteries, whereas groups or single microspheres were found in even more peripheral pulmonary artery branches.

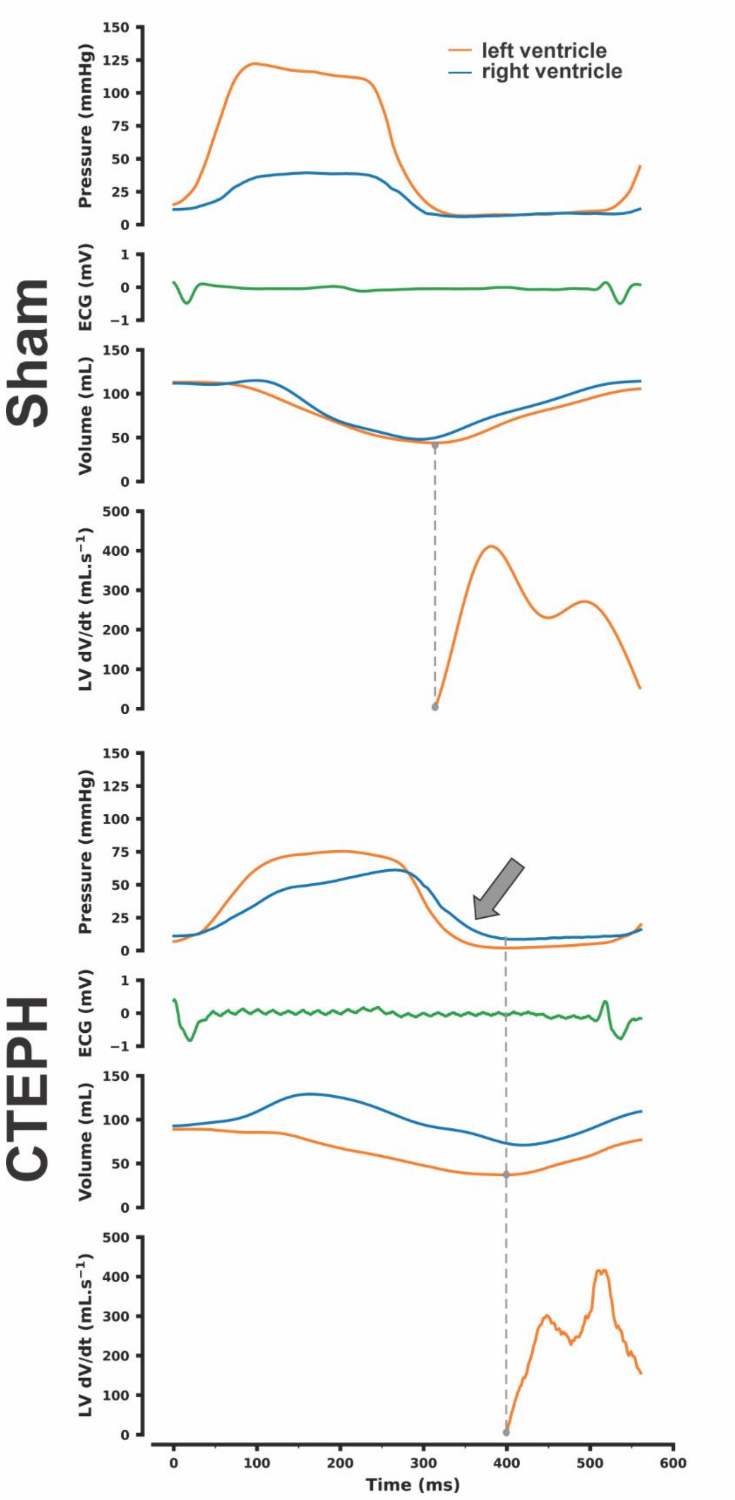

Supplementary Figure 5. Delayed early left ventricular filling illustrated by biventricular joint pressure-volume recordings. Representative time synchronized left and right ventricular pressure and volume signals from 1 beat in Sham and chronic thromboembolic pulmonary hypertension (CTEPH). Electrocardiography and transmitral filling as reconstituted from the derivative of left ventricular volume are also plotted. Please note that in a closed-chest preparation prolonged RV relaxation and

persistent elevation of pressures (arrow) lead to delayed and impaired left ventricular filling. This is likely the hemodynamic counterpart of rapid leftward septal shift at early diastole and parallel ventricular interdependence.

1 Supplementary Video Legends

Supplementary video 1. Bench side demonstration of the technique for suture deployment into the pulmonary arteries.

Supplementary video 2. Representative pulmonary artery angiographies. Top frames illustrate the left pulmonary artery (LPA) before (pre) and after (post) embolization intervention, middle frames the same for the right (RPA, and the bottom frame the outcome after the third intervention. Duration of individual videos was synchronized.

Supplementary video 3. Representative cardiac ultrasound clips in Sham and CTEPH terminal evaluation. Sham frames are presented on the left and CTEPH on the right, top frames were obtained with a phased-array cardiac ultrasound probe 2-chamber cross-sectional and 4-chamber longitudinal views are depicted, bottom frames were obtained after sternotomy with a linear-array (12MHz) probe placed on the epicardium, a 2-chamber cross-sectional view and a longitudinal view at the right ventricle flow tract are presented. Please note right ventricular hypertrophy and dilation as well as rapid leftward septal shift at early diastole. Duration of individual videos was synchronized.
